# Lost gallstones during laparoscopic cholecystectomy as a common but underestimated complication—case report and review of the literature

**DOI:** 10.3389/fsurg.2024.1375502

**Published:** 2024-04-09

**Authors:** L. Danhel, A. Fritz, L. Havranek, T. Kratzer, P. Punkenhofer, A. Punzengruber, D. Rezaie, S. Tatalovic, M. Wurm, R. Függer, M. Biebl, P. Kirchweger

**Affiliations:** ^1^ Department of Surgery, Ordensklinikum Linz, Linz, Austria; ^2^ VYRAL, Linz, Austria; ^3^ Department of Diagnostic and Interventional Radiology, Ordensklinikum Linz, Linz, Austria; ^4^ Medical Faculty, Johannes Kepler University, Linz, Austria

**Keywords:** spilled, lost, gallstones, laparoscopic cholecystectomy, abscess, case report, systematic review

## Abstract

**Introduction:**

Laparoscopic cholecystectomy (LC) represents one of the most commonly performed routine abdominal surgeries. Nevertheless, besides bile duct injury, problems caused by lost gallstones represent a heavily underestimated and underreported possible late complication after LC.

**Methods:**

Case report of a Clavien-Dindo IVb complication after supposedly straightforward LC and review of all published case reports on complications from lost gallstones from 2000-2022.

**Case Report:**

An 86-year-old patient developed a perihepatic abscess due to lost gallstones 6 months after LC. The patient had to undergo open surgery to successfully drain the abscess. Reactive pleural effusion needed additional drainage. Postoperative ICU stay was 13 days. The patient was finally discharged after 33 days on a geriatric remobilization ward and died 12 months later due to acute cardiac decompensation.

**Conclusion:**

Intraabdominal abscess formation due to spilled gallstones may present years after LC as a late complication. Surgical management in order to completely evacuate the abscess and remove all spilled gallstones may be required, which could be associated with high morbidity and mortality, especially in elderly patients. Regarding the overt underreporting of gallstone spillage in case of postoperative gallstone-related complications, focus need be put on precise reporting of even apparently innocuous complications during LC.

## 1 Introduction

Gallstone disease affects up to 20% of the European population. Laparoscopic cholecystectomy (LC) is indicated in patients with symptomatic gallstones, acute cholecystitis or biliary sludge and represents one of the most commonly performed abdominal surgeries (1).

Perforation of the gallbladder is relatively common in LC and is reported in various studies to range between 10% to 40% of procedures. Gallstone spillage is less common, and the true frequency of unremoved stones is difficult to determine. Some case series indicate a range of 6% to 30% (2). Incidence increases if the surgery is performed for acute cholecystitis. Other risk factors include male sex, higher age, obesity and the presence of postoperative adhesions. Complications resulting from these spilled stones are reported to occur in 0.08% to 0.3% of patients, and most of these lost stones remain clinically silent (2).

However, even if dropped gallstones do not cause actual postoperative harm through complications, they often are not correctly identified by imaging and can be mistaken for peritoneal lesions leading to unnecessary concern. Nevertheless, a small percentage of dropped gallstones cause actual complications of immediate or delayed (even months after surgery) clinical concern, such as abscesses and fistulas (3).

Some reports show that only half of the surgeons inform the patient when gallstones are lost during operation, less than 30% inform the general practitioner about this complication and less than a quarter of surgeons informed about this complication in the consent form handed to the patient preoperatively (4). Another part of the problem is the differentiation between intraoperative iatrogenic gallbladder perforation, spillage of gallstones, retrieved and lost gallstones. Underreporting of intraoperative gallbladder perforation is common and it is almost impossible to determine the exact number of spilled gallstones. Despite examination and rinsing, it may be impossible to assure, that all gallstones spilled into the abdomen are really retrieved.

We report a case of an elderly patient presenting with a symptomatic perihepatic abscess 6 months after LC.

## 2 Case report

An 86-year-old male patient presented in our surgical ward 6 months after presumed, uncomplicated laparoscopic cholecystectomy performed in May 2022 due to necrotizing cholecystitis with 15 kg of weight loss, anorexia and rapid feeling of fullness since the operation. The patient denied pain or fever. Upon physical examination, the patient reported diffuse abdominal discomfort. The abdomen was described as soft with mild tenderness in the right upper abdomen. Blood tests revealed elevated C-reactive protein and white blood count. His past medical history was significant for severe tricuspid valve insufficiency, atrial fibrillation, type 2 diabetes mellitus and arterial hypertension.

**Figure 1:**
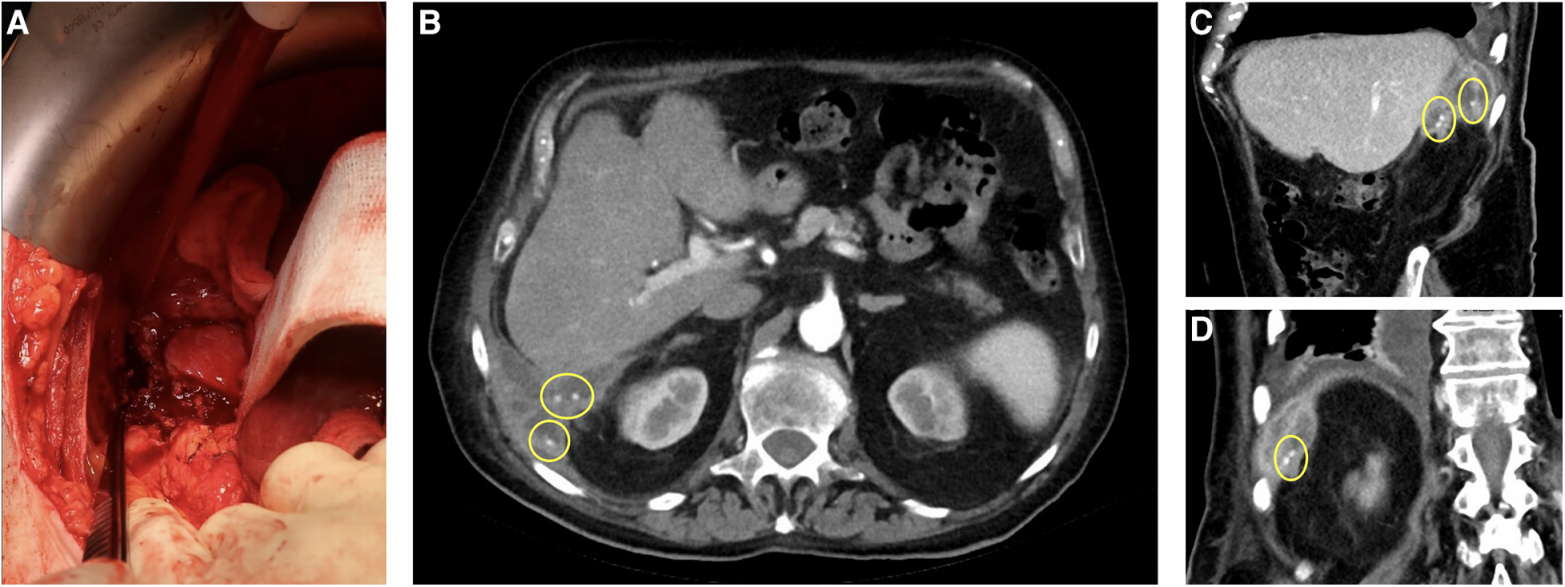
Computed tomography (CT) scan and intraoperative shot of the subhepatic abscess. (**A**) Macroscopic intraoperative image of the subhepatic abscess. (**B,C**) Subhepatic abscess in transverse and sagittal sections in CT scan, the lost gallstones are marked. (**D**) Subhepatic abscess in coronal section in CT scan, perforated diaphragm, right-sided pleural effusion, the lost gallstones are marked.

Computed tomography scan (CT, Figure 1) revealed a perihepatic abscess (5.5 × 5.8 cm) with suspected connection to the pleural space and small calcareous structures. Diagnostic laparoscopy was performed. Due to a soft, vulnerable liver, small liver injuries and bleeding, open surgery was necessary to successfully and safely drain the abscess. Upon evacuation, lost gallstones were discovered and removed. Further, the diaphragm was eroded by the chronic inflammation, but the parietal pleura was intact. Follow-up x-rays revealed an increasing pleural effusion, which was considered reactive. Therefore, the placement of a chest tube in the 5th intercostal space at the midaxillary line was additionally needed and was left for 4 days. Empirical intravenous antibiotic therapy with piperacillin + tazobactam 4,000 mg/500 mg twice a day for 3 days was initiated and then switched to meropenem as a single 1,000 mg dose once every 24 h due to increasing C-reactive protein. Antibiotics were de-escalated to cefuroxime 750 mg once a day after 4 days according to the antibiogram of the detected Escherichia coli isolated from the intraoperative swab. This antibiotic regimen was followed for another 6 days.

Postoperative ICU stay was 13 days. Reintubation was necessary due to cardiac decompensation with pulmonary edema. In addition, acute to chronic kidney failure developed with need for hemodiafiltration. Cardiac recompensation was achieved using Levosimendan and Landiolol.

The patient was finally discharged after additional 33 days on a geriatric remobilization ward, where his autonomous ability and everyday skills were restored. However, chronic kidney failure with need for hemodialysis persisted. The patient died 12 months after being discharged due to acute cardiac decompensation.

## 3 Discussion

Review of the literature resulted in 211 articles, and 89 records with 102 patients (5–92) were included in the analysis (Table 1). The median age was 62 years (IQR 29–87). However, age was not reported in 6 articles. In total, there were 37 (44%) male and 47 (56%) female patients. Gender could not be determined in 18 articles. Of all 102 reports, LC was performed as emergency procedure in 33 cases (32%) (7, 13, 19, 20, 24–27, 31–36, 38, 40, 42, 43, 47, 52, 53, 60, 63, 70, 73, 79–82, 84). In 20 articles, the indication for LC was not reported. Of all 102 case reports with lost gallstones, gallstone spillage had only been recorded by the surgeon in the surgical report in 31 cases (30%). The most commonly reported symptoms of symptomatic spilled gallstones were pain (*n* = 58, 56.8%), fever (*n* = 23, 22.5%), abdominal swelling (*n* = 18, 17.6%), weight loss (*n* = 11, 10.7%) and nausea or vomiting (*n* = 11, 10.7%). Other symptoms were fistulation (such as bronchobiliary, colovesical or atmospheric fistulas), night sweats, changes in stool, malaise, chills, gynecological complaints and also respiratory problems such as cough, hemoptysis or dyspnoea. Furthermore, pruritus, painless jaundice, urinary tract infection or gastrointestinal reflux have been described in individual cases. In 12 patients, lost gallstones were discovered as an incidental finding in asymptomatic patients (7, 10, 11, 19, 22, 40, 47, 61, 64, 68, 84). No symptoms were reported in 11 patients. Symptom onset was reported at a median of 36 months after surgery and ranged between 1 and 180 months. Postoperative abscesses caused by spilled gallstones were reported in 60/102 (58.8%) patients. Of these, 41.1% (*n* = 42) were intra-abdominal abscesses, 10.7% (*n* = 11) abdominal wall abscesses, 7.8% (*n* = 8) retroperitoneal abscesses and 6.8% (*n* = 7) lung abscesses. In 8 (7.8%) cases the lost gallstones mimicked malignancy. Lost gallstones may either mimic peritoneal carcinomatosis or the presence of a primary tumor, leading to excision (7, 22, 25, 32, 61, 84, 86). Remarkable 66.6% (*n* = 68) of the patients required open surgical procedures, 17.6% (*n* = 18) laparoscopic revisions and 12.7% (*n* = 13) were treated with ultrasound or CT guided drainage. Only 2 (1.9%) patients were successfully treated conservatively (53, 61).

**Table 1:** Study characteristics of all articles included.

#	Author, country	Year	Age	Gender	Indication for LC	Reference to the spilled stones	Presenting symptoms	Time of onset of symptoms after LC	Complications caused by lost stones and location found	Type of reintervention
1	McNamee, USA (58)	2022	57	M	NR	NR	Left lower quadrant abdominal pain	Several years	Inflammatory response in left lower quadrant	Laparoscopic removal
2	Almslam, Saudi Arabia (8)	2022	34	M	NR	NR	Abdominal pain, weight loss, night sweats	4 years	Inflammatory mass in the hepatorenal space	Robotic exploration
3	McCarley, USA (57)	2022	78	NR	NR	NR	NR	3 months	Subhepatic abscess	Percutaneous abscess drainage
4	Waleed, USA (85)	2022	44	M	NR	NR	NR	3 years	Peri-hepatic abscess	Open abscess-drainage with resection of portions of liver and diaphragm
5	Al-Janabi, Syria (7)	2022	54	F	Acute cholecystitis	NR	none	10 years	Mimicking intraabdominal tumor	Omentectomy during hysterectomy with bilateral salpingo-oophorectomy
6	Al-Janabi, Syria (7)	2022	29	F	Acute cholecystitis	NR	none	3 years	Mimicking intraabdominal implants	Resection during elective Caesarean section
7	Weeraddana, USA (86)	2022	66	F	Symptomatic cholelithiasis	NR	Right upper quadrant (RUQ) pain	5 years	Mass in the retroperitoneum behind the hepatic flexure mimicking a retroperitoneal Tumor	Surgical removal of the mass
8	Kendera, USA (48)	2022	70	F	NR	NR	RUQ pain, occasional nausea and vomiting	1 year	Perihepatic abscess	Percutaneous drainage
9	Tokuda, Japan (80)	2022	66	F	Acute gangrenous cholecystitis	Yes	RUQ pain, right chest pain and dyspnea	11 months	Pleural empyema and perihepatic fluid collection	Thoracotomy and laparotomy with gallstone retrieval
10	Zeledón-Ramirez, Costa Rica (92)	2022	62	F	NR	NR	RUQ pain, feverish feeling	3 months	Subcapsular hepatic abscess	Laparoscopic removal
11	Zeledón-Ramirez, Costa Rica (92)	2022	71	F	Elective laparoscopic cholecystectomy.	NR	Right flank pain	6 months	Right flank abscess	Percutaneous drainage
12	Fung, USA (31)	2022	69	M	Gangrenous cholecystitis	NR	Right-lower-quadrant abdominal pain, bloody diarrhea	5 months	Subhepatic Abscess	Laparosopic drainage
13	Kumar, USA (52)	2022	86	M	Acute cholecystitis	NR	Abdominal swelling, weight loss, nausea, emesis, loose stools	15 years	Abdominal Wall Abscess	Percutaneous drainage
14	Hoshina, USA (41)	2022	59	F	NR	NR	RUQ discomfort	2 years	Subdiaphragmatic abscess	Laparoscopic aspiration
15	Nagata, Japan (63)	2022	73	M	Acute cholecystitis	Yes	Fever, right chest pain, wet cough, and hemoptysis	6 months	Lung Abscess following Subphrenic Abscess	Thoracatomy with resection of Segment VIII in the lower lobe of the right lung, abscess drainage and retrieval of the dropped gallstone
16	Danhel, Austria	2022	86	M	Necrotizing cholecystitis	Yes	Weight loss, anorexia, reduced general condition	6 months	Perihepatic abscess with Connection to the pleural space	Laparotomy and drainage
17	Ray S, India (74)	2021	48	M	NR	NR	Right upper abdominal pain, low-grade fever and swelling on the site of the axillary port	39 months	Parietal wall abscess	Surgical removal
18	Mehmood, UK (59)	2021	65	M	Symptomatic cholelithiasis	Yes	Long standing dry cough, fever and painful swelling over the back in the right paraspinal area	8 years	Large abscess in the right paraspinal region and retroperitoneal abscess	Incision and drainage
19	Guruvaiah, USA (36)	2021	61	M	Acute cholecystitis	Yes	1-year history of intermittent RUQ pain, recurrent bronchitis and pneumonia with mucopurulent cough and sputum since his LC	Recurrent pneumonia since his LC	Bronchobiliary fistula	Trans-diaphragmatic takedown of the Fistula and right hepatic middle lobe wedge resection
20	Djelassi, Belgium (27)	2021	82	M	Perforated necrotic cholecystitis	NR	Chronic fistula at the RUQ	8 years	Abscess between the right internal oblique and transverses abdominis	Fistulectomy and drainage
21	Tchercansky, Argentina (79)	2020	69	M	Gallbladder empyema	Yes	Thoracic pain, cough and fever	5 months	Loculated pleural effusion of the Right Hemithorax in posterior cost-diaphragmatic recess	CT guided thoracic drainage initially and then Lung decortication by Video Assisted Thoracoscopy
										
22	Kafadar, Turkey (45)	2020	42	F	NR	NR	Painful swelling in suprapubic region persistent for 3 days	10 years	Omental granuloma	Partial omentectomy
23	Marçal, Portugal (56)	2020	79	F	Symptomatic cholelithiasis	NR	Painful right lumbar mass	3 years	Right subcutaneous lumbar abscess	Surgical drainage
24	Bolat, Turkey (19)	2020	62	M	Acute Cholecystitis	NR	4-year history of swelling of both right and left groins	5 months	Incidental finding in the right inguinal hernial sac	Surgical excision
25	Heywood, Australia (40)	2019	70	M	Emergency LC	NR	Incidental finding in the right inguinal hernial sac	5 years	Incidental finding in the right inguinal hernial sac	Surgical excision
26	Cummings, USA (24)	2019	70	M	Emphysematous cholecystitis and liver abscess	Yes	vague abdominal discomfort	2 years	Sub hepatic abscess	Surgical exploration + drainage
27	Akhtar, Pakistan (6)	2018	78	M	NR	NR	Recurrent bouts of abdominal pain and fever for the previous 2 weeks in the RUQ	10 years	19 cm Right subdiaphragmatic and retroperitoneal abscess	CT-guided drainage
28	Tyagi, USA (81)	2018	70	F	Acute Cholecystitis	Yes	Septic shock with fevers, chills, lethargy, altered mental status, right hip pain and an inability to move her hip or leg	2 months	Iliopsoas abscess and periprosthetic hip infection	Surgical drainage
29	Capolupo, Italy (22)	2018	73	M	Chronic cholecystitis	Yes	Peritoneal nodule detected during follow up for kidney stones	16 months	Mimicking peritoneal carcinomatosis	Laparoscopic excision
30	Urade, Japan (82)	2018	68	M	Gangrenous Cholecystitis	Yes	Left upper abdominal pain	7 months	Omental abscess and ascites around the spleen	Laparoscopic partial omentectomy and abscess drainage
31	Ologun, USA (66)	2018	52	F	Biliary colic	Yes	Occasional postpranding epigastric pain	4 years	Calcified intraabdominal mass within the omentum detected in routine follow up for laparoscopic sleeve gastrectomy	Laparoscopic resection of the mass
32	Stroobants, Belgium (77)	2018	72	F	Symptomatic cholelithiasis	NR	Intermittent complains about RUQ pain	NR	Subhepatic abscess	Open drainage
33	Kaplan, Israel (46)	2018	74	M	NR	NR	Six months vague RUQ pain	10 years	Perihepatic abscess	Laparoscopic drainage
34	Kaplan, Israel (46)	2018	41	F	NR	NR	One-month vague RUQ pain	3 years	Perihepatic abscess	Laparoscopic drainage
35	Koichopolos, Canada (51)	2017	80	M	Biliary disease	NR	Gastric outlet obstruction, 30 pounds weight loss, progressively worsening nausea, vomiting and significant gastroesophageal reflux	5 years	bulky circumferential irregular thickening and enhancement of the gastric wall at the level of the pylorus	Billroth II distal gastrectomy
36	Canna, UK (21)	2017	79	F	Chronic cholecystitis	NR	Painful and firm mass on the right flank	5 years	Retroperitoneal abscess	Surgical drainage
37	Lentz, USA (54)	2017	57	M	Symptomatic cholelithiasis	NR	Cough and right flank pain	2 years	Perihepatic, pulmonary and renal abscesses	Thoracic drainage
38	Faour, Syria (30)	2017	44	F	Symptomatic cholelithiasis	NR	Mass in the RUQ associated with pain, nausea and early satiety for the last 6 months	6 years	Intra-abdominal cystic mass	Surgical excision
39	Ragozzino, Italy (72)	2016	63	M	Chronic cholecystitis	NR	Intermittent vague discomfort of RUQ	2 years	Subphrenic abscess	Laparotomy, 3 × 3 cm mass excised
40	Kim, Korea (49)	2016	59	M	NR	NR	Constant RUQ pain	5 months	Retroperitoneal abscess	Laparotomy, 5 × 5 cm retroperitoneal mass was excised
41	Goodman, USA (32)	2016	87	F	Acute Cholecystitis	NR	Right flank pain and tenderness	4 years	Right flank soft tissue tumour extending into the abdominal wall	Surgical excision
42	Moga, Romania (60)	2016	66	F	Acute Cholecystitis	NR	Fever and large abscess in the right lumbar region	4 years	Right lumbar region abscess and subhepatic abscess	Laparoscopic drainage
43	Bedell, USA (16)	2015	41	F	Symptomatic cholelithiasis	NR	Dysmenorrhea progressed to chronic pelvic pain unrelated to menses	9 years	Pelvic abscess	Laparoscopic drainage
44	Binagi, USA (18)	2015	58	M	Symptomatic cholelithiasis	NR	Continuous but waxed and waned pain, reaching levels eight out of ten of Likert scale	3 years	Perihepatic abscess	Laparoscopic drainage
45	Grass, Switzerland (18)	2015	75	M	Acute cholecystitis	NR	Recurrent subcutaneous abdominal wall abscess with occasional, spontaneous drainage of pus	3 years	Abdominal wall abscess in the periumbilical port site	Surcical excision and drainage
46	Noda, Japan (64)	2014	52	NR	Symptomatic cholelithiasis	NR	Incidental US finding during medical check up	7 months	Subhepatic abscess	Percutaneous abscess drainage
47	Noda, Japan (64)	2014	41	NR	Symptomatic cholelithiasis	NR	RUQ pain	13 months	A rounded mass in the subhepatic space	Open drainage
48	Ahmad, UK (5)	2014	37	F	Symptomatic cholelithiasis, incidental pT1a gallbladder cancer	Yes	Recurrent RUQ pain	2 years	Multiple tumour embedded gallstones on the diaphragm and lesion in segment VI of the liver	Surgical excision of diaphragmatic nodules and liver segmentectomy VI
49	Lee, Korea (53)	2013	65	M	Recurrent acute cholecystitis	Yes	NR	7 months	Subhepatic abscess	Laparotomy, drainage
50	Lee, Korea (53)	2013	55	F	Gangrenous cholecystitis	Yes	NR	18 months	Cul de sac abscess	Laparotomy, drainage
51	Lee, Korea (53)	2013	48	F	Recurrent acute cholecystitis	Yes	NR	31 months	Umbilical fistula	Prolonged wound care
52	Lee, Korea (53)	2013	72	F	Gangrenous cholecystitis	Yes	NR	4 months	Right flank portal fistula	Prolonged wound care
53	Lee, Korea (53)	2013	80	M	Recurrent acute cholecystitis	Yes	NR	2nd post-operative day	Peritonitis	Antibiotic administration
54	Morris, USA (62)	2013	71	F	NR	NR	Pulmonary complains, diffuse abdominal pain, associated with nausea and emesis lasted for 24 h	15 years	Ileocolic torsion and cecal volvulus	Laparotomy, ileocecectomy
55	Peravali, UK (70)	2013	61	M	Acute cholecystitis	Yes	12-month history of persistent RUQ pain, 8 KG weight loss, anorexia, night sweats, intermittent pyrexical episodes	3 years	Sub hepatic abscess	Laparoscopic drainage
56	Peravali, UK (70)	2013	86	M	Acute cholecystitis	Yes	Chronically discharged right back fistula	5 years	Subphrenic abscess with atmospheric fistula	Lap drainage
57	Dobradin, USA (28)	2013	82	M	Elective cholecystectomy	NR	RUQ pain lasting for 2 months	8 years	Right flank abscess	Incision and drainage
58	Chatzimavroudis, Greece (23)	2012	72	F	Symptomatic cholelithiasis	Yes	High fever, chills and constant pain in the Right lumbar region for 2 days	6 months	Retroperitoneal abscess	CT-guided drainage
59	Gorospe, Spain (34)	2013	63	M	Acute cholecystitis	NR	Fever, malaise, weight loss	6 weeks	Fever of unknown aetiology	NR
60	Anrique, Chile (10)	2013	60	NR	NR	NR	Incidental finding during Lap Gynaecologic procedure	14 years	Multiple gallstones incrusted in the Douglas’ pouch	Surgical removal
61	Arai, Japan (11)	2012	65	M	Symptomatic cholelithiasis	NR	Abnormal liver mass detected on ultrasonography during a periodic medical check-up	4 years	Subphrenic abscess	Partial resection of the liver and right diaphragm
62	Papadopoulos, Greece (68)	2012	86	F	NR	NR	Incidental finding during right hemicolectomy	8 years	Gallstones embedded in the omentum	Laparotomy, Removal during right hemicolectomy
63	Singh, USA (75)	2012	42	F	NR	NR	Worsening right-sided tenderness and pain, low grade fever, night chills, weight loss	7 years	Subhepatic retroperitoneal inflammatory abscess	Laparotomy, Surgical excision of 4 × 6 cm
64	Rammohan, India (73)	2012	50	M	Calculous cholecystitis	NR	Minimally painful, slow progressing mass in the RUQ for the last two years	4 years	10 × 5 cm organised extrahepatic mass in the sub-diaphragmatic space extending onto the soft tissues of parietal wall	Laparoscopic piecemeal excision
65	Kayashima, Japan (47)	2011	57	F	Acute cholecystitis	Yes	Incidental abdominal US showed 3 liver lesions	3 years	Inflammatory pseudotumour of the liver	Posterior segmentectomy and concomitant resection of the diaphragm
66	Hussain, Saudi Arabia (43)	2010	33	NR	Acute cholecystitis	Yes	Intermittent attacks of RUQ pain, nausea, vomiting for 7 months	9 years	Discharging abdominal wall abscess extending to the retroperitoneum	Incision and drainage
67	Pottakkat, India (71)	2010	NR	F	Symptomatic cholelithiasis	NR	Fever, malaise, tender right subcostal swelling	11 years	Dumbbell abscess in the perihepatic area	Open drainage
68	Bouasker, Tunesia (20)	2010	57	F	Acute cholecystitis	NR	Inflammatory painful swelling of the right renal fossa	8 years	Subcutaneous collection and cutaneous fistula	Excision + Drainage, laparoscopic excision of the fistulous tract
69	Gooneratne, New Zealand (33)	2010	54	NR	Acute cholecystitis	NR	Recurrent urinary tract infections	14 years	Colovesical fistula	Surgical repair of the fistula
70	Helme, UK (39)	2009	77	F	NR	NR	Night sweets, right back pain and loin swelling for 2 weeks	5 years	Complex subphrenic, subhepatic and subcutaneous abscesses	US-guided drainage. Patient declined operation to remove the offending gallstones
71	Morishita, Japan (61)	2009	67	NR	Symptomatic cholelithiasis	NR	Incidental finding during FU for aneurysm	1 year	Granuloma mimicked malignancy	Conservative treatment
72	Dasari, UK (25)	2009	67	F	Acute cholecystitis	NR	Recurrent lower abdominal pain	2 years	Nodules mimicking peritoneal metastases	Laparoscopic excision
73	Maempel, UK (55)	2009	42	F	Symptomatic cholelithiasis	NR	Suspicious of strangulated recurrent paraumbilical hernia	10 years	Abdominal wall abscess	Incision and Drainage
74	Hougård, Denmark (42)	2008	64	F	Acute cholecystitis	Yes	Fistulas on the abdomen	7 years	Atmospheric fistula	Surgical excision
75	Arishi, Saudi Arabia (12)	2008	45	F	Symptomatic cholelithiasis	NR	Central colicky abdominal pains and swelling lasted for 6 months	15 years	Cystic mass of the rectus abdominis	Surgical excision
76	De Hingh, Netherlands (26)	2007	41	W	Acute cholecystitis	Yes	Abdominal pain and purulent vaginal discharge	1 year	Rectovaginal pouch abscess	Surgical excision
77	Stupak, USA (78)	2007	72	F	NR	Yes	Fever, nausea, anorexia, and pain in the RUQ lasting for 3 weeks	11 years	Subhepatic collection	Percutaneous drainage
78	Pantanowitz, USA (67)	2007	53	F	Symptomatic cholelithiasis	NR	Pelvic pain	7 years	Left ovar granuloma	Surgical excision
79	Wehbe, Australia (87)	2007	80	NR	Symptomatic cholelithiasis	NR	Abdominal pain, nausea, diarrhoea	10 years	Mass in the right lower quadrant	Laparoscopic excision
80	Wittich, USA (89)	2007	42	F	Symptomatic cholelithiasis	NR	Severe metrorrhagia, dysmenorrhea	13 months	Abscess in the pouch of Douglas	16 gallstones discovered after transvaginal hysterectomy
81	Bhati, UK (17)	2006	52	F	Symptomatic cholelithiasis	NR	Upper abdominal pain	1w	Liver abscess	Open drainage
82	Bhati, UK (17)	2006	60	F	Symptomatic cholelithiasis	NR	Fever and pain in her back	28 months	Subhepatic abscess	Open drainage
83	Bhati, UK (17)	2006	56	NR	Symptomatic cholelithiasis	NR	Fever and pain of the upper abdomen	7 years	Subdiaphragmatic abscess	Incision and Drainage
84	Ianniti, USA (44)	2006	70	M	NR	NR	Generalised aches and pains	3.5 years	Subphrenic and pleural abscess	Open and US guided drainage, due to recurrence open removal
85	Hand, USA (44)	2006	50	F	Biliary pancreatitis	NR	Pain, fever, large fluctuant mass lateral to umbilicus	2 years	Abdominal wall abscess	US-guided drainage, later local exploration and excision of the abscess
86	Viera, Italy (84)	2006	72	NR	Symptomatic cholelithiasis	NR	Fever, general malaise and weight loss	18 months	3 inflammatory lesions in Segment II and VII of the liver	Open excision
87	Viera, Italy (84)	2006	70	NR	Acute cholecystitis	Yes	Patient asymptomatic, incidental US finding	2 months	Hyperechoic images with posterior shadowing were observed in the Morison pouch	Watch and see approach
88	AlSamkari, USA (84)	2004	36	NR	Symptomatic cholelithiasis	Yes	Diffuse abdominal pain, nausea, vomiting and weakness	11 years	Necrotic transverse colon from mid-ascending to just distal the splenic flexure	Surgical excision
89	Koç, Turkey (50)	2004	75	M	Symptomatic cholelithiasis	NR	NR	6 years	Retroperitoneal abscess	Percutaneous drainage
90	Stevens, USA (76)	2003	68	F	Biliary pancreatitis	NR	Severe pruritus, nausea, painless jaundice, 30-pound weight loss and acholic stools	1 year	Subhepatic abscess	Open drainage
91	Aspelund, Iceland (76)	2003	NR	NR	Acute cholecystitis	NR	Symptomatic groin hernia	10 days	Gallstones in the hernial sac	Removal during hernia repair
92	Papasavas, Greece (69)	2002	77	F	Symptomatic cholelithiasis	Yes	Fever, pain	15 months	Right flank abscess	Surgical removal
93	Yadav, India (90)	2002	NR	NR	Symptomatic cholelithiasis	NR	NR	1 year	Subphrenic abscess	Open drainage
94	Van Mierlo, Netherlands (83)	2002	48	NR	Symptomatic cholelithiasis	Yes	Pain in the RUQ, nausea, vomiting	2 years	Subhepatic abscess	Open drainage
95	Hawasli, USA (38)	2002	75	F	Symptomatic cholelithiasis	NR	Pain, fever	4 years	Abdominal wall abscess	Open drainage
96	Hawasli, USA (38)	2002	43	M	Acute gangrenous cholecystitis	NR	Pain, fever	2 years	Subdiaphragmatic and subhepatic abscesses	NR
97	Famulari, Italy (29)	2002	NR	NR	Symptomatic cholelithiasis	NR	Dysuria, pollakiuria, vesical tenesmus	2 years	Urinary bladder granuloma	Partial cystectomy
98	Werber, USA (88)	2001	64	F	Symptomatic cholelithiasis	Yes	Low-grade fever with chills, night sweats, weight loss, fatigue	1 month	Sub hepatic abscess and 3 cm round mass with speculated borders in the right lower lobe of the lung	Right thoracotomy
99	Yao, China (88)	2001	NR	NR	Symptomatic cholelithiasis	NR	NR	2 years	Periumbilical abscess	Surgical excision
100	Battaglia, Italy (14)	2001	39	F	Symptomatic cholelithiasis	NR	Fever and pain	9 years	Abdominal wall abscess	Surgical excision
101	Ok E, Turkey (65)	2000	NR	NR	Symptomatic cholelithiasis	NR	NR	3 months	Incisional umbilical port site hernia	Surgical excision
102	Bebawi, USA (65)	2000	56	M	Chronic cholecystitis	Yes	Painful swelling of the right groin that was reducible before, and reducible swelling of the left groin	2 months	Gallstones in the hernial sac	Removed during hernia repair
TOTAL			Median 62 (29–87)	37 M, 47 F, 18 NR	33 acute cases, 20 NR	31 yes, 71 NR	Most prevalent: Pain 58, Fever 23, Swelling 18, Nausea/vomiting 11, weight loss 11, none 12	Median 36 months (1–180)	Total abscesses 60, intraabdominal abscesses 42, retroperitoneal abscesses 8, abdominal wall abscesses 11, lung abscesses 7, mimicking malignancy 8	Open procedure 68, laparoscopic procedure 18, ultrasound or CT drainage 13, watch and see approaches 2

M, male; F, female; RUQ, right upper quadrant; NR, nonreported; LC, laparoscopic cholecystectomy; KG, kilogram.

We aimed to conduct a census of all cases with complications from lost gallstones after laparoscopic cholecystectomy from 2000 to 2022 reported in the literature. The results should clarify that late complications from spilled gallstones are rare (0.08% to 0.3% of patients) but can cause severe problems that occur at a median of 36 months after the initial operation. However, it should be taken into consideration, that the published literature mainly covers incidental findings and small case series.

Of note, only 32% of reported cases initially had acute cholecystitis, while in the majority of cases, primary LC had been reported as elective procedure for symptomatic gallstone disease. Concerning a concept of a culture of safe cholecystectomy, surgeons should be facile with the following aspects: Knowledge of relevant anatomy, various anatomical landmarks, and anatomical variations; correct gallbladder retraction; safe use of energy devices; knowledge of the critical view of safety (including its documentation); awareness of various bailout procedures (e.g., cholecystectomy by the fundus-first approach) in difficult gallbladder cases; use of intraoperative imaging techniques (e.g., intraoperative cholangiogram) at uncertain anatomy; respecting the concept of time-out and thorough documentation (93).

It is also alarming that iatrogenic peroration of the gallbladder was only described in 30% of cases causing postoperative complications, suggesting a much higher number of actual gallbladder perforations during LC. Literature on incidental gallstone spillage may be biased by distinct underreporting, considering that only a minority of surgeons document gallbladder perforation and gallstone spillage. Mullerat et al. reported that only half of the surgeons informed their patients and less than 30% informed the general practitioner if gallstones were lost during surgery. The supposed low importance of these complications is underlined by the fact that only a quarter of the surgeons mention this complication in the surgical explanation (4). Operative difficulty is classified according to Nassar Grade and was found to be a significant independent predictor of 30-day complications and 30-day reinterventions. The score could be used to unify the severity of the disease and the technical difficulty of the operation and can be implemented as a tool to document operative findings. Therefore, it can be used in future research to compare outcome and intraoperative difficulties (94).

Since almost 60% of all complications are abscesses, predominant symptoms are fever, pain, abdominal swelling, weight loss, nausea or vomiting. This should lead to radiological cross-sectional imaging in acute diagnostics, which should quickly lead to the correct diagnosis.

The formation of an abscess can be life-threatening. The percutaneous placement of a drain or catheter under imaging control is an increasingly used medical procedure. It is an effective and safe alternative to surgery, reducing discomfort and hospitalization. An amazing 66.6% of the cases required an open procedure and only 12.7% of the patients could be treated with percutaneous drainage (95). Apparently retained gallstones are a problem for percutaneous techniques, because the removal of stones is complex or even impossible. Variations in the location of retained stones, clinical symptoms and individual risk factors of patients demand a personal treatment strategy. However, minimal invasive techniques should be applied, whenever appropriate. Thus, it remains questionable whether a standardized procedure can be found for this complication. In particular, confusion with peritoneal masses can have severe consequences. Complex symptoms such as gastrointestinal reflux, urinary tract infections or breathing problems may lead to a diagnostic dilemma.

## 4 Limitations

A collection of case reports has several limitations. As Gavriilidis et al. described, institutional, national, underpowered sample size, learning curve, performance and follow-up bias may have influenced the results. In addition, case reports with a poor outcome, unusual history of the disease and rare complications are more commonly reported in the literature, than those with an uncomplicated course (96).

One way to prevent these biases could be the implementation of international databases that record all complications of laparoscopic cholecystectomy postoperatively and in the follow-up. Therefore, awareness of this complication must be created. Futhermore, there is still a lack of a standardized procedure at the international level for laparoscopic operations for gallbladder diseases. Therefore, the Global Evaluation of Cholecystectomy Knowledge and Outcomes (GECKO) study (GlobalSurg 4) will be an international collaborative initiative that will allow contemporaneous data collection on the quality of cholecystectomies. GECKO is a prospective, international, multicentre cohort study observing patients undergoing cholecystectomy, between 31st July 2023 to 19th November 2023, with follow-up at 30-day and one-year postoperatively. The aim of this study is to define the global variation in compliance to pre-, intra-, and post-operative audit standards including: Interventional radiology service; risk stratification via Tokyo Guidelines 18; timing of surgery; achieving a critical view of safety; intraoperative imaging; initiating different bailout procedures; antibiotic use; use of drains; bile duct injury; 30-day readmission; and critical care (97).

## 5 Conclusion

This case report and review of the literature shall emphasize the alertness on exact reporting of complications to patients and attending doctors by exact documentation in operating reports, to think of that late complication after LC when the symptoms described above are present, and is simply intended to create general awareness, since many surgeons are probably not aware of the problem. Radiologists may suspect unclear radiopaque concretions in the CT scan as lost gallstones after LC in order to identify the abscess genesis earlier. It should be avoided that lost stones will not be considered in patients with above presented symptoms, as there is not a single note in the operation report about them being spilled.

Surgical management in order to completely evacuate the abscess and remove all spilled gallstones should be the attempted. Generally, laparoscopic approaches must be preferred for accessible abscess collection. However, percutaneous drainage could be considered as bridge to surgery or for patients unfit for surgery. Nevertheless, attempting to treat intra-abdominal abscesses containing spilled gallstones with percutaneous drainage will always bear the risk of incomplete treatment by leaving stones in the abdomen. If gallstones spill intraoperatively during laparoscopic cholecystectomy, all stones should be recovered and copious peritoneal lavage should be performed. The initial administration of antibiotics seems to be of secondary importance, as it seems most important to eliminate the mechanical trigger.

To sum up, most lost gallstones remain clinically silent, but they may cause complications that can become symptomatic after years from surgery. In patients with unexplained abdominal abscess or fistula with a history of cholecystectomy within the last 10 years, lost gallstones should always be considered.

## Data Availability

The original contributions presented in the study are included in the article/Supplementary Material, further inquiries can be directed to the corresponding author.
